# *Apis mellifera* as a Model Species to Evaluate Toxicological Effects of Fungicides Used in Vineyard Agroecosystems

**DOI:** 10.3390/jox15010018

**Published:** 2025-01-21

**Authors:** Tommaso Campani, Ginevra Manieri, Ilaria Caliani, Agata Di Noi, Silvia Casini

**Affiliations:** 1 Department of Physical, Earth and Environmental Sciences, University of Siena, Via Mattioli 4, 53100 Siena, Italy; 2 Santa Chiara Lab, Università di Siena, Via Val di Montone, 1, 53100 Siena, Italy

**Keywords:** pollinators, pesticides, biomarkers, toxicological effects, agroecology

## Abstract

Agroecosystems provide habitats, food, and water for many pollinators and insects, but they are also heavily exposed to threats from the widespread use of pesticides and fertilizers. Managed honeybees and wild bees encounter pesticides in vineyards by collecting morning dew from vine leaves and accessing sugars from grapes, particularly during dry periods. This study assessed the toxicological effects of the commercial fungicide formulations Fantic FNCWG^®^ and Ramedit combi^®^, both individually and in combination, on honeybees. Using a multi-biomarker approach, we evaluated neurotoxicity, metabolic disturbances, phase II detoxification processes, and immune system function. Our findings revealed that commercial fungicide mixtures with multiple active ingredients affect bees differently than single active compounds. Biomarker responses highlighted how these complex mixtures disrupt various enzymatic pathways; including immune function; altering critical enzyme kinetics involved in detoxification and potentially impairing essential bee functions. This study emphasizes the need for more comprehensive research into the sublethal effects of commercial pesticides, particularly those used in vineyards, which are understudied compared to pesticides used in orchards.

## 1. Introduction

The factors behind the loss of honeybees are many, and climate change, habitat destruction, the increase in diseases and parasites [1,2], and exposure to heavy metals and pesticides are the most important [3,4]. This last category of compounds is a threat of great relevance to bees’ health, especially due to intensive agriculture, which led to a significant increase in the use of pesticides in recent years [5]. The study of the negative effects of plant protection products on honeybees has always focused on those substances that directly come into contact with them such as insecticides, widely used in orchards, fungicides and herbicides [6,7,8] and, more generally, the substances that act on flowers were bees feed. On the other hand, it is increasingly important to study the effects of substances widely used in crops where pollinators are not typically expected to be present, such as in vineyards [9].

Several European regions are famous for wine production, with many hectares of land covered by vineyards as a monoculture. During droughts, when blooms are scarce, vineyards become vital feeding grounds for bees, with honeybees gathering morning dew from vine leaves and drawing sugar and water from the grapes.

These behaviors are accentuated by continuous climate change and can cause the exposure of bees to a group of plant protection products little investigated. The main treatments applied in a grapevine are fungicides, among which copper and sulfur are most frequent [10], but synthetic compounds also find frequent applications [11]. Copper-based fungicides, such as Bordeaux mixture, are widely used to control the downy mildew disease of grapevine cultivation [12,13]. Copper has been used in viticulture for more than 150 years, at rates of up to 80 kg ha^−1^ per year, which has led to the accumulation of copper in the topsoil of many vineyards [14]. The use of copper fungicides in organic agriculture is currently restricted in Europe [15]. To date, the amount of copper intensively used in organic farming is limited to 4 kg ha^−1^ per year in most European countries, including Italy, France, and Spain [16]. Used in combination with copper, the cymoxanil is a broad-spectrum, systemic fungicide used to protect various fruits, vegetables, and field crops from a wide range of fungal diseases and is particularly effective in controlling downy mildew [17,18]. It belongs to a class of aliphatic nitrogen compounds and functions as a foliar fungicide with both protective and curative effects. The literature about the toxicological effects of cymoxanil on bees or other insects is absent, and the literature about the effects on vertebrates is scarce. According to a study by Ahmed et al. [19], cymoxanil significantly increased levels of alkaline S-transferase (AST), alanine transaminase (ALT), and alkaline phosphatase (ALP) in the serum and liver of mice, suggesting tissue necrosis and potential leakage of these enzymes into the bloodstream.

The work of Chang et al. [20] suggested that famoxadone-cymoxanil can induce developmental toxicity, immunotoxicity and neurobehavioral toxicity in zebrafish larvae.

Another fungicide widely used since the fifties in the vineyard is folpet. Folpet is an organochlorine phthalimide with a molecular weight of 296.6 g/mol and is used as a protective, broad-spectrum fungicide against leaf spot diseases in grapevines. It acts by reacting with thiols, altering proteins and enzymes in fungi [21]. By nonspecific reaction with thiols, folpet reacts with cysteine amino acids in proteins and glutathione, thus affecting the function of many proteins and enzymes, altering honeybee worker gene expression and disturbing energy production with a reduction in foraging activity and hormonal dysregulation, such as the transition of nurse bees to foragers [22]. Moreover, folpet was found to induce cytotoxicity and tumorigenic activity in the gastrointestinal tract in mice [23] and mortality in larval stages of *Rana temporaria* and *Bufotes viridis* [24].

Folpet active principle studied by Canal-Raffin and collaborators [25] in the commercial formulation, Folpan 80WG^®^, showed a cytotoxic effect on human bronchial epithelial cells in vitro that could be in part explained by oxidative stress, lipid peroxidation and antioxidant activity.

The ecotoxicological approach based on biomarkers permits highlighting the sublethal effect caused by exposure to a specific contaminant or a mix of known or unknown contaminants. Biomarkers are a powerful tool for assessing the sub-lethal effects before irreversible damage occurs to organisms and colonies [26]. Alterations at a lower biological level can serve as important early warning signals to prevent macroscopic effects at the ecological level [26].

Our study aimed to investigate the toxicological effects on honeybees, used as model species, of the commercial formulation of two fungicides: FanticFNCWG^®^ (folpet-based) and Ramedit combi^®^ (cymoxanil + copper-based), alone and in combination. A multi-biomarker approach was applied to test neurotoxicity, measuring acetylcholinesterase (AChE) and carboxylcholinesterase (CaE) activities, metabolic alterations with alkaline phosphatase (ALP), the phase II of the detoxification process with glutathione S-transferase (GST), and the efficiency of the immune system by evaluating lysozyme (LYS) and phenoloxidase (proPO, PO) activities.

## 2. Materials and Methods

### 2.1. Chemicals

All the following chemicals were obtained from Sigma-Aldrich (St. Louis, MO, USA): monobasic and dibasic sodium phosphate, sodium chloride (NaCl), PBS, Tritons X-100, protease inhibitor cocktail powder; acetylthiocholine iodide (AcSCh.I), 5,5-dithio-bis(2, nitrobenzoic acid) (DTNB); 1-chloro-2,4-dinitrobenzene (CDNB), reduced L-glutathione (GSH); α-naphthyl acetate (αNA) and α-naphthyl butyrate (αNB); tris-hydroxy-methyl-aminomethane (Tris), magnesium chloride (MgCl_2_), p-nitrophenyl phosphate (p-NPP); *Micrococcus lysodeikticus* solution, egg whites from chicken (HEL); monobasic potassium phosphate and bovine serum albumin (BSA), α-chymotrypsin, L-DOPA. BioRad Protein stain was obtained from BioRad (Segrate, Italy), while Diff-Quick dye was from Bio-optica (Milano, Italy). FANTIC F NCWG^®^ was purchased from Gowan Italia S.r.l. (Faenza, Italy), and Ramedit combi^®^ from Isagro S.p.A (Faenza, Italy).

### 2.2. Laboratory Exposure Condition

The laboratory experiments were carried out using worker honeybees about three days old that came from a synchronized brood from the same hive. During the experiment honeybees were fed with sucrose solution (50%) with the addition of treatments; the control consisted of only sucrose solution (50%). The feeding rate measured during the administration of the treatments and the mortality rate were checked daily. Honeybees were exposed to Fantic F NCWG^®^ at 1 g/L (F1) and 2 g/L (F2), Ramedit combi^®^ at 0.5 g/L (R0.5) and 1 g/L (R1), and the mix of the two (MIX1: F1 + R0.5 MIX2: F2 + R1) fungicides. The higher concentration used corresponded to the field application rates indicated on the label. The exposure was carried on for 6 days, following the previous protocol used [26]. To avoid a high mortality rate caused by captivity, the first day of the experiment was used for the acclimatation of bees. From the second day, each group of treated honeybees (25 bees in 2 cages for each treatment) was fed with a total of 12 mL of the treatment dissolved in sugar syrup, administered on alternate days.

The commercial fungicide Fantic F NCWG^®^ composition was benalaxyl-m 3.75% and folpet 48.00% active ingredient and other coadjuvants not reported on the label, while Ramedit Combi^®^ composition was cimoxanil 4.2% + metallic copper (in the form of copper oxychloride 39.75%) and other coadjuvants not on the label.

### 2.3. Samples Preparation and Enzyme Assays

At the end of the experiment, bees were anesthetized in ice (4 °C). The midgut was then removed with small forceps, and the head was separated from the rest of the body. All the samples were immediately frozen and stored at −80 °C. The brain was used to evaluate esterase activity (AChE and CaE). Metabolic and biotransformation enzymes (GST, ALP) and lysozyme activity (immune system) were evaluated on midgut extracts following the methodologies by Caliani and collaborators [26]. PO and proPO activities were evaluated in the thorax according to Burciaga and collaborators [27].

Tissue samples were pooled and weighed, and extraction medium was added in a volume corresponding to 10% (wt/vol) of the tissue. The buffer contained 40 mM Na phosphate buffer (pH 7.4), a mixture of protease inhibitors, and 1% Triton X-100. The samples were homogenized by a tissue lyser homogenizer (Qiagen, Hilden, Germany) for three periods of 30 s at 30 s intervals. After the homogenates were centrifuged at 4 °C for 20 min at 13,000× *g* for head and gut samples. The thorax and wings were removed, and together with the abdomen, they were homogenized in PBS and subsequently homogenized by a tissue lyser homogenizer (Qiagen) for three periods of 30 s at 30 s intervals. After the homogenization, the samples were centrifuged at 4 °C at 15,000× *g* for 15 min.

### 2.4. Pesticide Interaction Model

We applied the Pesticide Interaction Model according to Almasri et al. [28]. The IR (interaction ratio) considers the mortality rate of bees in the control group with respect to the treatment groups. The model obtained allows the identification of two different toxicological behaviors: the additive IR ≥ 1 or the synergistic IR > 1 effect. When the IR is <1, we have four other types of interaction: (i) purely antagonistic interaction when the mixture has a lower effect in comparison with the lowest toxic substance; (ii) sub-additive interaction, when the mixture has an effect higher than the most toxic substance but below the expected mortality; (iii) sub-additive synergistic effect when the toxicity of the mixture falls between the effects of the least toxic substance and the most toxic substance; or (iv) the effect of the mixture was considered independent when it caused a mortality rate similar to that of each pesticide individually [28].

### 2.5. Statistical Analysis

We tested significant differences in each biomarker between the control group, the fungicide FANTIC F NCWG^®^, the fungicide Ramedit Combi^®^, and the mixture samples using the Kruskal–Wallis (KW) non-parametric test. We applied Dunn’s test with a Benjamini–Hochberg stepwise adjustment for pairwise multiple comparisons when the null hypothesis of the KW test was rejected. Statistical differences in food consumption were tested using Student’s *t*-test. All analyses and graphical representations were implemented with RStudio (2023.06.09) and R (version 4.2.2).

## 3. Results

### 3.1. Laboratory Exposure

**Figure 1:**
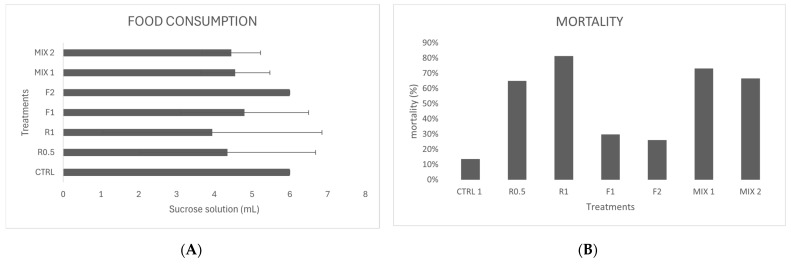
Feeding rate (mean and standard deviation (**A**)) and mortality (**B**), of the honeybees exposed to control (CTRL), fungicides Fantic^®^(F1, 1 g/L and F2, 2 g/L), Ramedit^®^ (R0.5, 0.5 g/L and R1, 1 g/L), and two mixes of both fungicide (MIX1: F1 + R0.5; MIX2 F2 + R1).

The feeding and the mortality rate are reported in Figure 1A,B. Sugar syrup intake was monitored after administering the treatment dose, with measurements taken for a total of two inputs. Mortality was recorded cumulatively after the experiment.

All the treatments showed a reduction in food intake in the second input with respect to the control without a statistical difference between the inputs (*t*-test 1.97; *p* = 0.096). However, all the experimental groups had feeding rate values not lower than 66%. The feeding results confirm the full intake of the fungicides Fantic^®^ and Ramedit^®^ from the honeybees exposed.

The mortality rate of honeybee specimens exposed to the fungicides Fantic^®^ and Ramedit^®^ is presented in Figure 1. These data are necessary to apply the Pesticide Interaction Model. Following Almasri et al. [12], the higher toxicity of a compound is associated with higher mortality, so in order of toxicity: R1 (81%) > R0.5 > (65%) > F1 (30%) > F2 (26%). The results obtained show that concerning MIX1 and MIX2, we are in a sub-additive interaction of the third case.

### 3.2. Biomarkers Results

**Figure 2:**
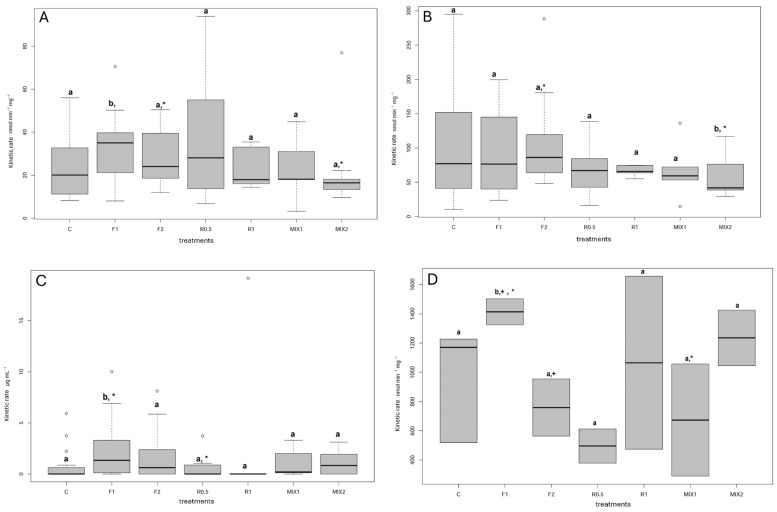
Activities of GST (**A**), ALP (**B**), LYS (**C**), and CaE (**D**) measured in honeybees (*Apis mellifera*) exposed to two different doses of Fantic^®^ (F1; F2) and Ramedit^®^ (R0.5; R1) and related mixtures (MIX1; MIX2)**.** Different lowercase letters indicate significant differences with respect to control (*p* < 0.05); the same symbols (”*”, “+”) indicate significant differences between the group marked (*p* < 0.05).

The results obtained from the evaluation of GST (Kruskal–Wallis (K-W): Χ^2^ = 15.3107, *p* = 0.02) (Figure 2A) in specimens of *A. mellifera* exposed in the laboratory, showed higher values of activity after the exposure to Fantic^®^ (F1), compared to the control group (*p* = 0.0313). On the other hand, a decrease in GST activity was observed in specimens exposed to the MIX2 compared to the bees exposed to F1 (*p* = 0.0128) and F2 (*p* = 0.048).

The results of ALP activity (K-W: Χ^2^: 16.7213, *p* = 0.01) (Figure 2B) showed lower values in MIX2 with respect to the control (*p* < 0.024) and between MIX2 and F2 (*p* < 0.0497) with lower values in MIX2.

The LYS activity (K-W: Χ^2^: 19.8143, *p* = 0.000) (Figure 2C) showed an induction after all treatments compared to the control, except for treatment R1, which showed strong inhibitions in the activity. In particular, statistically significant differences were found between F1 and the control (*p* = 0.0017) and between F1 and R0.5 (*p* = 0.0058) and R1 (*p* = 0.0299) treatments.

The phenoloxidase pathway PO and proPO ratio showed higher values in the F1 (*p* = 0.041) and F2 (*p* = 0.0035) treatment with respect to the control.

The results of AChE activity evaluated in the brain tissues of honeybees exposed to the fungicides and related mixtures did not show any variation or neurotoxic effect with a median average activity of 2.5 (nmol min^−1^ mg prot^−1^). Differently, the results of the CaE activity, evaluated in the same brain tissues of honeybees (Figure 2D), showed induction with a statistically significant difference between the F1 treatment and the control (*p* = 0.05). We also found a significant difference between the F1 and F2 (*p* = 0.0193) treatments, with a reduction in the activity from the lowest to the highest dose of the treatment. Moreover, we found statistically significant differences between F1 and MIX1 (*p* = 0.008) and between F1 and R0.5 (*p* = 0.0038). The CaE results for MIX1, composed of the sum of the F1 dose and R0.5 one, as well as for the GST data, confirmed the sub-additive effect in honeybees exposed to the mixtures.

## 4. Discussion

In our study we applied the Pesticide Interaction Model, proposed by Almasri et al. [28] and previously described, considering the mortality rate of bees to understand if the selected fungicides had additive, synergistic, or antagonistic effects on honeybees. The higher toxicity of a compound is associated with higher mortality, observing that R1 had the highest mortality rate, followed by R0.5, F1, and F2 (26%). The results obtained after the exposure to the mixtures of fungicides showed that we are in a sub-additive interaction of the third case. Almasri et al. [28] found the same interaction in *A. mellifera* exposed to the active ingredients of the fungicide difenoconazole (F), the insecticide imidacloprid (I), and the herbicide glyphosate (H) for 20 days.

The glutathione S-transferase is a class of enzymes whose primary role is to facilitate the conjugation of the antioxidant molecule glutathione (GSH) with various electrophilic and hydrophobic compounds, such as toxic substances like pesticides and reactive oxygen species (ROS). The increase in GST activity was observed by Caliani et al. [26] in specimens of honeybees topically exposed to concentrations of 100 g/L and 200 g/L of the fungicide AmistarXtra^®^. In the study by Han et al. [29] conducted on *Apis cerana*, the GST activity of newly emerged bees was reduced following exposure to the fungicide propiconazole, while no statistically significant difference in GST activity was found between the exposed groups and the control group for forager bees. The results obtained in the present work suggest that exposure to the fungicides Fantic^®^ and Ramedit^®^ may influence the detoxification activity of GST. It can both increase the activity compared to the control bees and inhibit the activity when mixed at a lower concentration.

Glutathione and other thiol-containing molecules play an essential role in the inactivation of the folpet active principle of Fantic^®^. It has been described that the presence of cytoplasmatic GSH reduces the folpet concentration in the tissues of several species [30]. Also, the reduction in GSH availability induced the activity of GST to regenerate GSH. Our results could highlight this mechanism in the exposure to Folpet-based fungicide.

Alkaline phosphatase is a class of enzymes that helps break down phosphate-containing nutrients vital for the insect’s growth and energy, such as nucleotides and phospholipids, into simpler forms that can be absorbed. It is also involved in the immune response, particularly in the gut. It can participate in the detoxifying process of toxins and assist in protecting the gut lining against harmful substances like pathogens. The ALP inhibition in honeybees exposed to the MIX1 and MIX2 compared to other treatments was probably due to the presence of copper in the Ramedit^®^ commercial fungicide. Since there was a statistical difference between F2 and MIX2, we assumed that Folpet was not responsible for the inhibition in the mix. Our study was the first study to investigate the toxicological effects on alkaline phosphatase of copper-based fungicides and cymoxanil. Until now, other studies have examined the effects on the ALP enzyme for different types of metals and fungicides: Vlahovic et al. [31] highlighted that the ALP activity was inhibited following exposure to cadmium. The study by Badiou-Beneteau et al. [32] on bees exposed to lower doses (2.56 ng bee^−1^) of the insecticide thiamethoxam showed a 20% increase in ALP activity compared to the control. Inhibition of ALP was also observed in the study by Caliani et al. [26] following exposure of *A. mellifera* specimens to concentrations of 100 g/L and 200 g/L of the fungicide AmistarXtra, concentrations of 0.1 and 2.5 g/L of cadmium, and a concentration of 12.4 g/L of EMS.

Lysozyme is synthesized in the fat body of honeybees [33] and plays an essential role in the innate immune system of insects, aiding in the defense against microbial infections, especially bacteria. Insects depend heavily on their innate immune responses, as they lack the adaptive immune systems found in vertebrates. Lysozyme is a useful biomarker for investigating disease resistance and therefore the immune system status of an organism. The study by Caliani et al. [26] exposed *A. mellifera* to the fungicide AmistarXtra (100 g/L and 200 g/L), cadmium (0.1 g/L and 2.5 g/L), and EMS (12.4 g/L), resulting in a statistically significant decrease in LYS activity compared to the control after both cadmium doses, the highest fungicide dose, and the EMS dose. These results were in line with the findings of our work, where bees exposed to copper-based fungicide caused a reduction in the immunological capacity of lysozyme. Differently, the folpet-based fungicides caused an induction of the lysozyme activity. Furthermore, LYS activity is negatively correlated with ALP activity in the experimental groups R0.5 (ρ = −0.46; *p* < 0.05) and F1 (ρ = −0.42, *p* < 0.05). As the activity of ALP increases, the activity of the immune system decreases, indicating that the detoxifying enzyme is working while the immune system is being taxed. LYS is also correlated with GST in the experimental groups exposed to the lowest (ρ = −0.52; *p* < 0.05) and the highest concentration (ρ = −0.44, *p* < 0.05) of Ramedit^®^, specifically indicating that as GST detoxifying activity increases, the functionality of the immune system decreases due to the presence of toxic substances in the honeybee’s body. Therefore, our results suggest that exposure to the fungicides Fantic^®^ and Ramedit^®^ causes an alteration in the immune system of bees, increasing lysozyme activity, especially following exposure to Fantic^®^ and to the mixtures of the two fungicides.

Another critical component of the immune system in invertebrates is the phenoloxidase pathway. It plays an essential role in insect defense against pathogens such as bacteria, fungi, and parasites [34]. The central enzyme in this pathway is prophenoloxidase (proPO), a zymogen of phenoloxidase (PO). Upon exposure to pathogens or injury, the proPO is activated into phenoloxidase (PO) by proteolytic cleavage [35]. This activation is usually mediated by serine proteases that are part of the immune response. Phenoloxidase catalyzes the oxidation of phenols to quinones that subsequently polymerize to form melanin, which is deposited around the invading pathogen [36]. The PO/proPO ratio indicates that efficiency increases as the value approaches one. The preliminary laboratory results of this biomarker obtained in this work showed the activation of PO in the specimens exposed to both the Fantic treatments. Similarly to lysozyme, exposure to Fantic triggers an increase in phenoloxidase, activating key components of the immune system.

The esterase enzymes, acetylcholinesterase and carboxylesterase, play key roles in the transmission of nerve impulses, with acetylcholinesterase being particularly crucial for this process. Carboxylesterase is a class of enzymes which are also involved in the detoxification of neurotoxic substances. The absence of neurotoxicity in all the treatments highlighted by the AChE results could be related to the induction of CaE, which plays a role in phase I detoxification processes and also serves a defensive function by protecting AChE from inactivation [37].

## 5. Conclusions

Laboratory experiments have demonstrated that the commercial formulation of fungicides, containing multiple active ingredients, produces different effects compared to exposure to individual molecules. By applying interaction models and conducting a multi-biomarker evaluation, we were able to more accurately explain the interactions between compounds when mixed. This approach allowed us to better replicate what occurs under environmentally realistic conditions. Biomarker responses revealed that these complex mixtures impact various enzymatic pathways, such as the immune system, altering fundamental enzyme kinetics involved in the detoxification process and potentially disrupting normal bee functions. This study underscores the need for a more thorough investigation of the sublethal effects of commercial pesticides, especially those used in vineyards, which are less studied compared to compounds applied in orchards. Such research is crucial for the protection of managed and wild pollinators, expanding our understanding of pesticide impacts and their role in the Colony Collapse Disorder (CCD) phenomenon.

## Data Availability

The raw data supporting the conclusions of this article will be made available by the authors on request.
